# Design of Thienothiophene-Based Copolymers with Various Side Chain-End Groups for Efficient Polymer Solar Cells

**DOI:** 10.3390/polym12122964

**Published:** 2020-12-11

**Authors:** Ying-Chieh Chao, Jhe-Han Chen, Yi-Jie Chiou, Po-lin Kao, Jhao-Lin Wu, Chin-Ti Chen, Li-Hsin Chan, Ru-Jong Jeng

**Affiliations:** 1 Institute of Polymer Science and Engineering, National Taiwan University, Taipei 10617, Taiwan; ejc0864381@gmail.com; 2 Department of Applied Materials and Optoelectronic Engineering, National Chi Nan University, Nantou 54561, Taiwan; s102328505@mail1.ncnu.edu.tw (J.-H.C.); ejejej247@gmail.com (Y.-J.C.); s106328029@mail1.ncnu.edu.tw (P.-l.K.); 3 Institute of Chemistry, Academia Sinica, Taipei 11529, Taiwan; dreamgroove@gmail.com (J.-L.W.); chintchen@gate.sinica.edu.tw (C.-T.C.)

**Keywords:** two-dimensional conjugated copolymer, benzodithiophene, thienothiophene, acceptor end groups

## Abstract

Three two-dimensional donor–acceptor conjugated copolymers consisting of a benzo[1,2-b:4,5-*b*′]dithiophene derivative and thieno[3,2-*b*]thiophene with a conjugated side chain were designed and synthesized for use in bulk heterojunction (BHJ) or nonfullerene polymer solar cells (PSCs). Through attaching various acceptor end groups to the conjugated side chain on the thieno[3,2-*b*]thiophene moiety, the electronic, photophysical, and morphological properties of these copolymers were significantly affected. It was found that the intermolecular charge transfer interactions were enhanced with the increase in the acceptor strength on the thieno[3,2-*b*]thiophene moiety. Moreover, a better microphase separation was obtained in the copolymer: PC_71_BM or ITIC blend films when a strong acceptor end group on the thieno[3,2-*b*]thiophene moiety was used. As a result, BHJ PSCs based on copolymer:PC_71_BM blend films as active layers exhibited power conversion efficiencies from 2.82% to 4.41%, while those of nonfullerene copolymer:ITIC-based inverted PSCs ranged from 6.09% to 7.25%. These results indicate the side-chain engineering on the end groups of thieno[3,2-*b*]thiophene unit through a vinyl bridge linkage is an effective way to adjust the photophysical properties of polymers and morphology of blend films, and also have a significant influence on devices performance.

## 1. Introduction

Solar cells have attracted great attention as the most promising alternative resource of energy. Among all kinds of solar cells, polymer solar cells (PSCs) have attracted extensive attention and become an emerging technology due to their benefits, such as light weight, low cost, easy solution processing, and potential for use in large-area flexible devices through roll-to-roll or printing technologies [1,2,3]. Typically, a PSC device structure comprises a conjugated polymer as an electron donor and a fullerene derivative [e.g., [6,6]-phenyl C_71_ butyric acid methyl ester (PC_71_BM)] as an electron acceptor and features nanoscale, phase-separated bulk heterojunction (BHJ) morphology between the two electrodes. Through the great efforts of many researchers, power conversion efficiencies (PCEs) of PSCs have attained over 13% for traditional single-junction PSCs and over 14% for tandem PSCs [4,5]. Nonfullerene acceptors have attracted much attention owing to their strong visible-near-infrared light-harvesting capability and tunable energy levels. In addition, they exhibit higher short-circuit current density (*J*_sc_) and PCE in PSCs than fullerene acceptors, leading to a PCE of 18% that was reported recently [6,7,8]. In most PSCs with high efficiency, the conjugated polymer donor material often contains electron-rich and electron-deficient units in the polymer backbone, creating a push-pull effect that results in intra- or intermolecular charge transfer (ICT) and gives rise to a polymer with a low bandgap and broad absorption band [9,10]. For building low-bandgap polymers, the benzo [1,2-*b*:4,5-*b*′]dithiophene (BDT) derivative is a promising unit owing to its excellent electronic properties [11,12] and have been developed by combining it with different acceptors, such as thieno [3,4-*c*]pyrrole-4,6-dione [13,14,15], quinoxaline [16,17,18], benzothiadiazole [19,20,21], diketopyrrolopyrrole [22,23,24] and thieno [3,4-*b*]thiophene (TT) [25,26,27]. Among these copolymers, a combination of BDT units and TT units has received much attention owing to the symmetric and planar structure of BDT and the stabilization of the quinodial structure from TT, which facilitates π-electron delocalization, improves π–π interactions, and narrows the energy gap of the resulting polymer [28,29,30]. Through molecular structure optimization by the Yu group, the side-chain substituent of the BDT skeleton comprised 2-ethylhexyloxy groups and the 3-position on the TT unit was given a fluorine atom, forming a polymer named PTB7. A high PCE of 7.4% was obtained from a PTB7/PC_71_BM-based PSC [31]. Chen et al. reported an analog polymer of PTB7 with fine modification of the chemical structure in which the 2-ethylhexyloxy group was replaced with an alkylthienyl group on the BDT unit. Constructing the polymer with two-dimensional (2D) conjugated side chains (designated as PTB7-Th) to lower the HOMO energy level and broaden the absorption range yielded a PCE of 7.4%, while device engineering modifications performed by Li et al. significantly increased the PCE to over 10% [32,33]. Later, Li et al. reported a new polymer (designated as PBDT-S-TT), with further fine modification, where they replaced the alkylthienyl substituents on the BDT unit with alkylthiothienyl groups, and the PCE reached 8.42%. The high PCE was attributed to the alkylthiothienyl groups which lowered the HOMO level and enhanced the hole mobility of the polymer [34]. In addition to replacing various substituents on the BDT unit, the modification on the TT unit was found to be another effective way to fine tune the electronic properties of synthesized polymers. Hou et al. used a sulfonyl group to replace the ester group on the 2-position of the TT unit to construct a polymer named PBDTTT-S, which showed a device PCE of 6.22% [35]. The sulfonyl group provided a strong electron-withdrawing effect that greatly influenced the LUMO level as well as the HOMO level, and this result suggests that it is possible to affect the overall electronic properties by introducing a strong electron-withdrawing group on a TT unit to improve the performance of PSCs. Peng et al. recently reported two new 2D polymers based on modified BDT and TT units, denoted as PBDTTT-CN and PBDTTT-S-CN. In these polymers, the BDT used alkylthienyl or alkylthiothienyl groups as substituents, while the TT utilized alkoxycarbonyl cyanovinyl groups as pendants linked by a vinyl bridge. The PBDTTT-S-CN-based PSCs showed a PCE of 7.00%, which was ascribed to the alkylthiothienyl and cyanovinyl substituents on the BDT and TT units, respectively. The substituents affected the electrochemical and optical properties of the polymer simultaneously, leading to broader absorption and lower energy levels than those of PBDTTT-CN and resulting in a high PCE [29].

Herein, on the basis of the above considerations, we design and synthesize three 2D donor–acceptor conjugated copolymers consisting of a BDT unit, with alkylthiothienyl substituents, and a TT unit, with a vinyl linkage attaching various acceptors as end groups, for use in PSCs. We add various electron-withdrawing groups as end groups via vinyl linkage on the TT unit to increase the strength of the acceptor. The various end groups were selected owing to their commercial availability and ease of construction on the TT unit via Knoevenagel condensation. Through a vinyl bridge linking the various accepting end groups to the TT unit, the electronic properties of TT moiety were directly adjusted. In this work, the influence of the accepting end groups on the TT moiety on the photophysical and photovoltaic properties of the constructing copolymers is investigated in detail. The morphological and photovoltaic characteristics of the copolymer/fullerene derivative or nonfullerene-blend films are discussed. This work demonstrates that various end groups in the copolymer structure significantly influence the absorption range, electronic energy levels, and morphologies of thin films and potentially serve as active-layer materials for PSC applications.

## 2. Experimental Details

### 2.1. Materials

All the reagents and solvents were purchased from Sigma-Aldrich, Alfa Aesar, and TCI Chemical Co., and used without further purification. Diethyl ether, *N*,*N*-dimethylformamide and xylenes were dried over appropriate drying agents and freshly distilled before use. 4,8-Bis(5-(2-ethylhexyl)thiophen-2-yl)benzo[1,2-*b*:4,5-*b*′]dithiophene-2,6-diyl)bis(trimethylstannane) (compound **9**) was synthesized according to the methods in the literature [36]. Poly(3,4-ethylenedioxythiophene):poly(styrenesulfonate) (PEDOT:PSS, Clevios P-VP AI4083) was purchased from Uni-onward corporation and used as received. 3,9-bis(2-methylene-(3-(1,1-dicyanomethylene)-indanone))-5,5,11,11-tetrakis(4-hexylphenyl)-dithieno[2,3-*d*:2′,3′-*d*′]-s-indaceno[1,2-*b*:5,6-*b*′]dithiophene (ITIC) and [6,6]-phenyl C_71_ butyric acid methyl ester (PC_71_BM) used for polymer solar cells fabrication were purchased from Sigma-Aldrich and Solenne, respectively.

#### 2.1.1. 4-Bromothiophene-3-carbaldehyde (**1**)

3,4-Dibromo thiophene (7.07 mL, 64 mmol) was dissolved in anhydrous diethyl ether (3 mL), and *n*-BuLi (40 mL, 64 mmol) dropwise added in the mixture at −78 °C under nitrogen atmosphere. After the reaction mixture was stirred for 30 min, *N*,*N*-dimethylformamide was slowly added to the mixture, and then stirred at −78 °C for 2 h. After, the reaction mixture was poured into water and extracted with diethyl ether. The combined organic layers were dried over MgSO_4_, and the solvent was removed by rotary evaporation. Further purification was conducted on a silica column using dichrolomethane:hexanes (1:2) as the eluent to yield compound **1** as brown viscous liquid. Yield: 8.8 g (70%). ^1^H NMR (300 MHz, CDCl_3_, ppm): 9.95 (s, 1H), 8.16 (d, 1H, *J* = 3.6 Hz), 7.37 (d, 1H, *J* = 3.3 Hz).

^13^C NMR (100 MHz, CDCl_3_, ppm): 184.25, 137.12, 134.59, 124.90, 110.81.

#### 2.1.2. Ethyl Thieno[3,4-*b*]thiophene-2-carboxylate (**2**)

4-Bromothiophene-3-carbaldehyde (6.69 g, 35 mmol), ethyl 2-sulanyl acetate (4.22 mL g, 38.5 mmol), copper oxide nanopowder (0.56 g, 7 mmol) and K_2_CO_3_ (7.26 g, 52.5 mol) were dissolved in dimethylsulfoxide (70 mL). The reaction mixture was heated at 80 °C in the nitrogen during for 16 h, and then the mixture was poured into ice water and extracted with ether. The combined organic layers were dried over MgSO_4_, and the solvent was removed by rotary evaporation and the desired product was purified by column on silica–gel using dichrolomethane:hexanes (1:2) as the eluent to give compound **2** as a pale yellow solid. Yield: 4.98 g (65%). ^1^H NMR (300 MHz, CDCl_3_, ppm): 7.69 (s, 1H), 7.58 (d, 1H, *J* = 2.4 Hz), 7.27 (d, 1H, *J* = 2.7 Hz) 4.36 (q, 2H), 1.38 (t, 3H). ^13^C NMR (100 MHz, CDCl_3_, ppm): 163.10, 145.92, 139.80, 123.41, 116.54, 111.31, 61.59, 29.67, 14.26.

#### 2.1.3. Thieno[3,4-*b*]thiophene-2ylmethanol (**3**)

Thieno[3,4-*b*]thiophene-2-carboxylate (0.67 g, 3.14 mmol) was dissolved in anhydrous ether (6.5 mL), and then LiAlH_4_ (2.67 g, 0.122 mol) was added portion-wise in the mixture. The reaction mixture was stirred for 2 h. After the reaction mixture was poured to 1 M NaOH organic layer was extracted with diethyl ether. The combined organic layer was dried over MgSO_4_, and the crude product was purified by column on silica–gel using dichrolomethane:hexanes 1:2 as eluent to give compound **3** as a white solid. Yield: 0.34 g (63%). ^1^H NMR (300 MHz, CDCl_3_, ppm): 7.27 (d, 1H, *J* = 2.7 Hz), 7.21 (d, 1H, *J* = 2.4 Hz), 6.84 (s, 1H), 4.78 (d, 2H, *J* = 6.3 Hz), 1.91 (t, 1H). ^13^C NMR (100 MHz, CDCl_3_, ppm): 150.77, 146.76, 138.77, 114. 28, 112.00, 110.85, 61.52.

#### 2.1.4. Thieno[3,4-*b*]thiophene-2carbaldehyde (**4**)

PCC (pyridinium chlorochromate) (2.74 g, 12.7 mmol) was slowly added to a mixture of thieno[3,4-*b*]thiophene-2-ylmethanol (1.84 g, 10.8 mmol) in dichloromethane (10.8 mL) at room temperature under an atmosphere of nitrogen for 16 h, and then the mixture was poured into water and the organic layer was extracted with ethylacetate. The combined organic layer was dried over MgSO_4_, and the solvent was removed by rotary evaporation. The crude product was purified by column on silica–gel using dichrolomethane:hexanes 1:2 as eluent to yield compound **4** as a yellow solid. Yield: 0.65 g (36%). ^1^H NMR (300 MHz, CDCl_3_, ppm): 10.00 (s, 1H), 7.76 (d, 1H, *J* = 2.7 Hz), 7.68 (s, 1H), 7.33 (d, 1H, *J* = 2.4 Hz). ^13^C NMR (100 MHz, CDCl_3_, ppm): 185.49, 149.75, 145.73, 139.34, 128.01, 118.93, 111.97.

#### 2.1.5. 4,6-Dibromothieno[3,2-*c*]thiophene-2-carbaldehyde (**5**)

To a solution of thieno[3,4-*b*]thiophene-2-carbaldehyde (1.35 g, 8.1 mmol) in CHCl_3_ (8.5 mL) and AcOH (8.5 mL), *N*-bromosuccinimide (3.20 g, 18.0 mmol) was added portion-wise at 0 °C, and the mixture thus formed was stirred in the nitrogen at room temperature for 12 h. The mixture was into iced water, extracted twice using dichloromethane, dried over anhydrous MgSO_4_ and evaporated to full dryness, and the desired product was purified by column on silica gel using hexane:ethyl acetate 10:1 as eluent to give compound **5** as a yellow solid. Yield: 1.42 g (54%). ^1^H NMR (300 MHz, CDCl_3_, ppm): 9.97 (s, 1H), 7.54 (s, 1H). ^13^C NMR (100 MHz, CDCl_3_, ppm): 184.83, 150.46, 145.58, 139.97, 127.24, 104.76, 98.03.

#### 2.1.6. 2-((4,6-Dibromothieno[3,4-*b*]thiophen-2-yl)methylene)malononitrile (**6**)

Malononitrile (24 mg, 0.36 mmol) and aluminum oxide (30 mg, 0.29 mmol) was dissolved in 2 mL dichloromethane and stirred for 1 h at room temperature under an atmosphere of nitrogen. Next, the above mixture was filtered and the solution was added slowly to a mixture of 4,6-dibromothieno[3,2-*c*]thiophene-2-carbaldehyde (30 mg, 0.09 mmol) in dichloromethane (2 mL). The reaction mixture was stirred at room temperature for 12 h. The mixture was poured into water and extracted with dichloromethane. The combined organic layers were dried with over MgSO_4_, and the solvent was removed by rotary evaporation. The desired product was purified by column (dichloromethane:hexane 1:4 as eluent) to afford compound **6** as a yellow-green solid. Yield: 35 mg (95%). ^1^H NMR (300 MHz, CDCl_3_, ppm): 7.78 (s, 1H), 7.40 (s, 1H). ^13^C NMR (100 MHz, CDCl_3_, ppm): 151.70, 144.64, 141.80, 140.04, 129.37, 113.19, 112.02, 105.77, 98.02, 82.70. EI-HRMS: calculated 373.8005, *m*/*z* = 373.7998 (M^+^).

#### 2.1.7. (E)-2-(benzo[*d*]thiazol-2-yl)-3-(4,6-dibromothieno[3,4-*b*]thiophen-2-yl)acrylonitrile (**7**)

2-Benzothiazoleacetonitrile (150 mg, 0.86 mmol) and aluminum oxide (150 mg, 1.47 mmol) was dissolved in 15 mL toluene and stirred for 1 h at room temperature under an atmosphere of nitrogen. Next, the above mixture was filtered and the solution was added slowly to a mixture of 4,6-dibromothieno[3,2-*c*]thiophene-2-carbaldehyde (150 mg, 0.46 mmol) in toluene (10 mL). The reaction mixture was heated at 110 °C in the nitrogen for 12 h. After the reaction mixture was cooled to room temperature, the mixture was poured into water and extracted with dichloromethane. The combined organic layers were dried with over MgSO_4_, and the solvent was removed by rotary evaporation. The desired product was purified by column (dichloromethane:hexane 1:4 as eluent) to afford compound **7** as an orange solid. Yield: 136 mg (61%) ^1^H NMR (300 MHz, CDCl_3_, ppm): 8.32 (s, 1H), 8.08 (d, 1H, *J* = 8.4 Hz), 7.93 (d, 1H, *J* = 7.8 Hz), 7.55 (t, 1H), 7.46 (t, 1H), 7.42 (s, 1H). ^13^C NMR (100 MHz, CDCl_3_, ppm): 161.50, 153.72, 145.38, 143.72, 140.10, 138.99, 135.37, 127.19, 126.29, 126.19, 123.73, 121.79, 115.85, 105.97, 102.80, 97.31. EI-HRMS: calculated 481.8039, *m*/*z* = 481.8026 (M^+^).

#### 2.1.8. (Z)-5-((4,6-dibromothieno[3,4-*b*]thiophen-2-yl)methylene)-3-ethyl-2-thioxothiazolidin-4-one (**8**)

The piperidine (3 drops) was added to a mixture of 4,6-dibromothieno[3,2-*c*]thiophene-2-carbaldehyde (150 mg, 0.46 mmol) and 3-ethylrhodanine (150 mg, 0.93 mmol) in chloroform (10 mL). The reaction mixture was heated to reflux for 8 h. After cooling to room temperature, the mixture was poured into water and extracted with dichloromethane. The combined organic layers were dried with over MgSO_4_, and the solvent was removed by rotary evaporation. The desired product was purified by column to afford compound **8** as a yellow solid. Yield: 155 mg (72%). ^1^H NMR (300 MHz, CDCl_3_, ppm): 7.74 (s, 1H), 7.09 (s, 1H), 4.21–4.16 (m, 2H), 1.29 (t, 3H). ^13^C NMR (100 MHz, CDCl_3_, ppm): 191.54, 166.99, 146.03, 144.60, 125.98, 125.18, 123.39, 101.67, 97.36, 40.07, 29.70, 12.23. EI-HRMS: calculated 468.7756, *m*/*z* = 468.7756 (M^+^).

#### 2.1.9. Synthesis of P1

A mixture of compound **6** (193 mg, 0.515 mmol) and compound **9** (500 mg, 0.515 mmol) were dissolved in 10.5 mL of anhydrous xylenes in a 10–20 mL microwave tube under a nitrogen atmosphere. The reaction mixture was degassed over 20 min at 50 °C, followed by addition of Pd(PPh_3_)_4_ (30 mg, 5 mmol%) in the tube. The mixture was stirred under microwave irradiation by 80 °C for 2 min, 130 °C for 2 min, 160 °C for 2 min, 185 °C for 2 min and 200 °C for 40 min, and then the reaction was quenched through the addition of aqueous potassium fluoride solution and toluene. The solvent was evaporated through vacuum distillation; the residue was dissolved in chloroform and filtered through Celite^®^ 545. The crude polymer was precipitated through dropwise addition of the chloroform solution into hot methanol. The solid residue was purified through successive Soxhlet extraction with hexane, methanol, acetonitrile, and acetone to removed monomers, oligomers, and impurities. Finally, the polymer was extracted into chloroform. The solution was concentrated under vacuum to give **P1** as a deep dark green solid with isolated yields of 31% after drying under a vacuum for 24 h. ^1^H NMR (300 MHz, CD_2_Cl_2_, δ/ppm): 8.00–6.11 (br, 8H), 3.55–2.41 (br, 16H), 1.82–0.40 (m, 14 H).

#### 2.1.10. Synthesis of P2

The synthetic procedure of P2 was similar to P1, using compound **7** (248 mg, 0.515 mmol), compound **9** (500 mg, 0.515 mmol) and Pd(PPh_3_)_4_ (30 mg, 5 mmol%) in 10.5 mL of anhydrous xylenes. Following the same purification methods as described for P1, the P2 was obtained as a dark green solid with an isolated yield of 77%. ^1^H NMR (300 MHz, CD_2_Cl_2_, δ/ppm): 7.85–6.32 (br, 12H), 3.55–2.45 (br, 16H), 1.82–0.40 (m, 14 H).

#### 2.1.11. Synthesis of P3

P3 was prepared using the same procedures as described for P1 using compound **8** (109 mg, 0.232 mmol) and compound **9** (225 mg, 0.232 mmol) in 4.7 mL of anhydrous xylenes. Following the same purification methods as described for P1, the P3 was obtained as a dark green solid with a yield of 80% after drying under a vacuum. ^1^H NMR (300 MHz, CD_2_Cl_2_, δ/ppm): 7.90–6.85 (br, 8H), 3.45–2.82 (br, 18H), 1.85–0.45 (m, 15 H).

### 2.2. Characterization of Copolymers

Microwave reactions were performed in sealed vessels using an Anton Paar microwave reactor (Anton Paar Monowave 300). ^1^H NMR and ^13^C NMR spectra were recorded on a Varian Unity Inova 300WB NMR spectrometer at room temperature. The molecular weights of copolymers were obtained by gel permeation chromatography (GPC) using a Waters GPC-1515 in tetrahydrofuran (THF) via polystyrene as the standard. Thermogravimetric analysis (TGA) was conducted on a Perkin-Elmer TGA-7 analyzer with a heating rate of 10 °C/min under nitrogen atmosphere, and the thermal decomposition temperatures (*T*_d_) of the copolymers were determined at which a 5% weight loss occurred. UV-Vis absorption spectra were measured on a Hewlett-Packard 8453 spectrophotometer. Cyclic voltammetry (CV) was conducted on a CHI 612D electrochemical workstation at a scanning rate of 50 mV s^−1^ in an anhydrous nitrogen-saturated solution of 0.1 M Bu_4_NClO_4_ in MeCN. A three-electrode cell was used, wherein a thin polymer film coated on a Pt plate was used as the working electrode, a Pt wire as the counter electrode, and Ag/Ag^+^ electrode as the reference electrode, respectively. The electrochemical potential was calibrated against Ferrecene/Ferrocene^+^. Surface morphologies of the active layers were performed by atomic force microscopy (AFM) system in the tapping mode using an SII SPA400 operating in ambient atmosphere and transmission electron microscopy (TEM) at an accelerating voltage of 120 kV using a JEOL JEM-1200EX II instrument.

### 2.3. Fabrication and Characterization of PSCs

All PSCs were prepared using the fabrication procedure as follows. The patterned indium tin oxide (ITO) glass with a sheet resistance of 8 Ω/sq. was cleaned sequentially with surfactant, deionized water, acetone and isopropyl alcohol. Following the cleaned glass substrates were dried on a hot plate, and treated with oxygen plasma for 5 min. For the conventional PSCs, the hole-transporting material poly(3,4-ethylenedioxythiophene):poly(styrenesulfonate) (PEDOT:PSS, Clevios P-VP AI4083) was passed through a 0.45 μm filter and spin-coated on the ITO-coated glass at a spinning rate 4000 rpm for 60 s. The sample thus formed was then placed on a hot plate and dried at 120 °C for 10 min. Subsequently, the copolymer/PC_71_BM layer (with an optimal weight ratio of 1:1.5, in a total concentration of 18 mg mL^−1^) in *o*-dichlorobenzene (*o*-DCB) was formed on the top of PEDOT:PSS layer by spin-coating (600 rpm, 60 s) the mixture solution and then annealed at 140 °C for 10 min. The Ca-based (30 nm)/Al (100 nm) cathode was deposited onto the photoactive thin film by thermal evaporation in a high-vacuum chamber and both at a controlled deposition rate of 1 Å s^−1^. For the inverted PSCs, the zinc oxide (ZnO) precursor was prepared by dissolving 250 mg of zinc acetate dihydrate and 70 mg of ethanolamine in 2.5 mL of 2-methoxyethanol. The precursor solution was then deposited on top of the ITO-coated glass and annealed at 200 °C for 1 h in air prior to use [37]. After that, the copolymer/ITIC layer (with an optimal weight ratio of 1:1, in a total concentration of 18 mg mL^−1^) in *o*-DCB was spin-coated (600 rpm, 60 s) on the top of ZnO/ITO and then annealed at 140 °C for 10 min. The MoO_3_ (7 nm)/Ag (100 nm) anode were deposited by thermal evaporation in a high-vacuum chamber and the evaporation rate of MoO_3_ and Ag were 0.5 Å s^−1^ and 1.0 Å s^−1^, respectively. The active area of the PSC was 4 mm^2^, which was defined by the overlap of the ITO and metal electrode. Following deposition of the cathode, the PSCs were examined under ambient conditions. Current density–voltage (*J-V*) characteristics of the PSCs were recorded using a Keithley 2400 source meter with simulated solar irradiation at an intensity of 100 mWcm^−2^ (AM 1.5G, Oriel solar simulator). The incident photon-to-electron conversion efficiency (IPCE) was measured by using a Si reference photodiode and a xenon lamp as the light source, and all the measurements were conducted in air.

## 3. Results and Discussion

### 3.1. Characterization of Copolymers

**Scheme 1:**
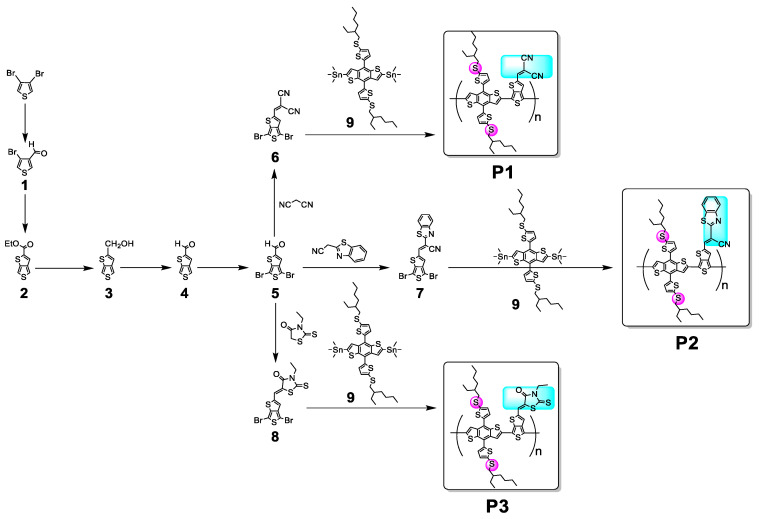
Synthetic scheme for P1–P3 copolymers.

**Figure 1:**
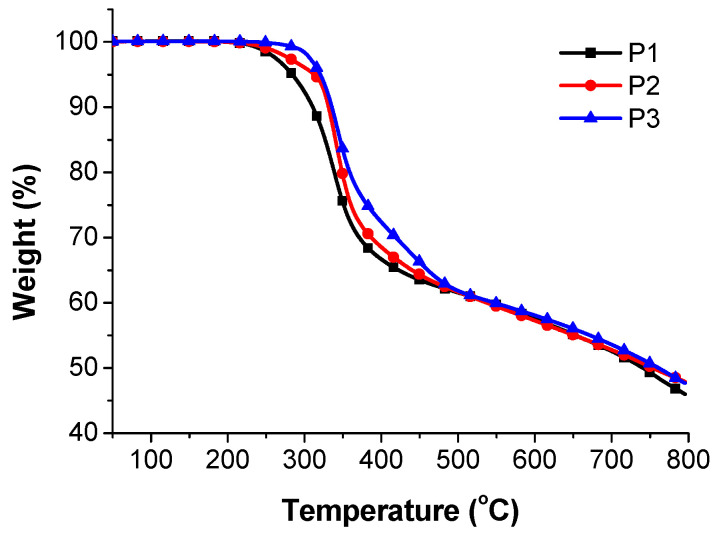
Thermogravimetric analysis (TGA) diagrams of the copolymers at a heating rate of 10 °C/min under nitrogen atmosphere.

The processes for synthesizing the three copolymers (P1, P2, and P3) are shown in Scheme 1. Compound **1** through Compound **5** were obtained according to the literature with some modifications [38]. Compound **5** was then reacted with malononitrile, 2-benzothiazoleacetonitrile, or 3-ethylrhodanine via Knoevenagel condensation to form compounds **6**, **7**, and **8** with yields of 95, 61, and 72%, respectively. Compound **9** was copolymerized with Compound **6**, Compound **7**, or Compound **8** via a Stille coupling reaction in equimolar ratios to form copolymers P1, P2, and P3, respectively. The obtained copolymers were precipitated in methanol and then purified by Soxhlet extraction to remove impurities. Table 1 presents the number-average (*M*_n_) and weight-average (*M*_w_) molecular weights of these polymers as determined by GPC against polystyrene standards with THF as the eluent. The *M*_w_ values of these polymers were in the range from 18.2 to 36.8 kDa, corresponding to the polydispersity index (PDI) values from 1.23 to 1.65. The thermal properties of these polymers were obtained by thermogravimetric analysis, and the thermal degradation temperatures (*T*_d_) were defined as the point at which a 5% weight loss occurred, as shown in Figure 1 and Table 1. The *T*_d_ values of samples P1, P2, and P3 were 285, 314, and 321 °C, respectively, under an atmosphere of nitrogen, which indicated these copolymers exhibited sufficient thermal stability for their application in PSCs.

### 3.2. Photophysical Properties

**Figure 2:**
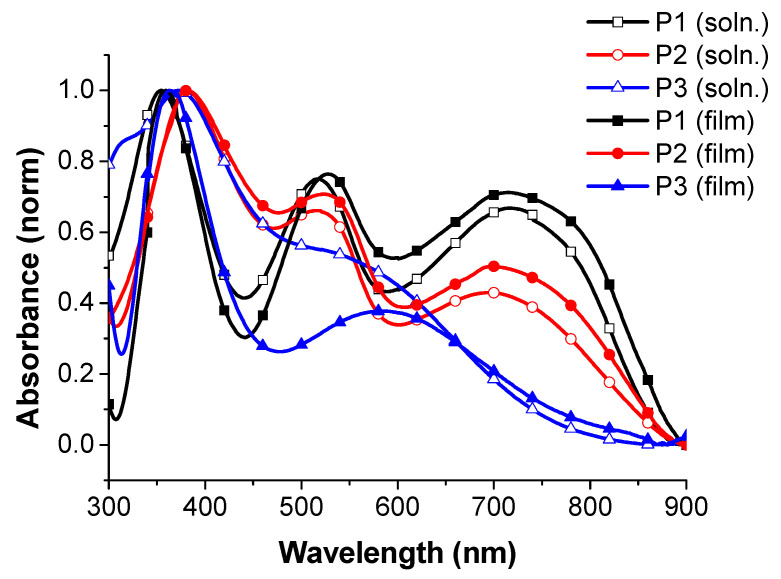
UV-visible absorption spectra of the copolymers in *o*-dichlorobenzene solutions and in thin films.

Figure 2 displays the UV-Vis absorption spectra of the copolymers in *o*-dichlorobenzene solutions and thin films, and Table 2 summarizes the corresponding absorption data of these copolymers. In solution, three copolymers exhibited broad absorption in the region of 300–900 nm and featured three clear absorption peaks except for P3. For P1 and P2, the absorption band in the range of 300–630 nm originated from conjugated side chains of BDT and the π–π* transition of the polymer backbone. The absorption band in the range of 630–900 nm was attributed to the ICT interactions between the donor (BDT unit) and acceptor (TT unit). In contrast with P1 and P2, P3 only showed two peaks in the absorption spectrum: the π–π* transition of the polymer backbone and the ICT interactions overlapped, which implied that the P3 ICT interactions were weaker than those of P1 and P2 [39]. In comparison with the spectra in solutions, the three copolymers were red-shifted and broadened in the thin film state, this implies that the π–π stacking interactions and ordered aggregation of the polymer chains were enhanced. The broad absorption is beneficial for increasing the sunlight harvesting ability and is expected to increase the absorption ability of the active layer. Moreover, the strong π–π stacking interactions and ordered aggregation are well suited for charge transport in devices, leading to an increase in the induced photocurrent in PSCs. The results herein suggest the photophysical properties of these copolymers are influenced by the side chains on TT moieties and can be tuned by manipulating the conjugated side chain-end groups of the TT units.

### 3.3. Energy Levels and Energy Gaps

**Figure 3:**
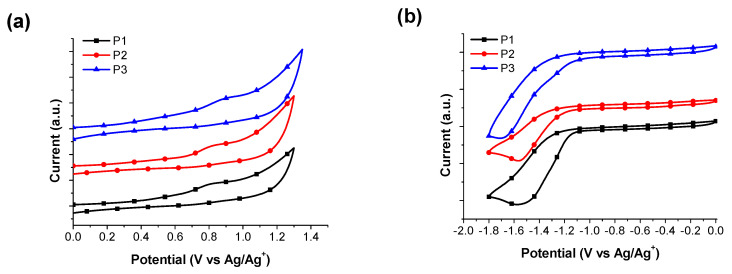
Cyclic voltammograms of copolymer films at (**a**) oxidation, and (**b**) reduction in acetonitrile solution.

The energy levels of these copolymers were analyzed by cyclic voltammetry (CV) experiments, and the HOMO and LUMO energy levels were estimated from the onset of the oxidation and reduction potential using ferrocene as the reference and assuming an absolute energy level of −4.8 eV for Fc/Fc^+^ in vacuum [40]. Figure 3 shows the characteristics of the copolymers that are evident from the CV measurements. The HOMO and LUMO values are also summarized in Table 2. As shown in Table 2, the HOMO levels of P1, P2, and P3 are −5.52, −5.50 and −5.60 eV, respectively, while the LUMO levels are −3.69, −3.65, and −3.72 eV, respectively. P1, P2 and P3 possessed the same donor moieties in the polymer main chain, but exhibited different HOMO energy levels, which implied the HOMO energy levels of these copolymers were affected by the end groups of the TT units through the conjugated system of polymer main chains. Apparently, the LUMO energy levels of these copolymers were almost determined by the acceptor strength of the end groups on the TT unit, since the only difference between these copolymers was the various acceptor end groups. The results implied the energy levels of these copolymers could be simply tuned by attaching different acceptor end groups onto the TT units, as evidenced by the photophysical properties.

**Figure 4:**
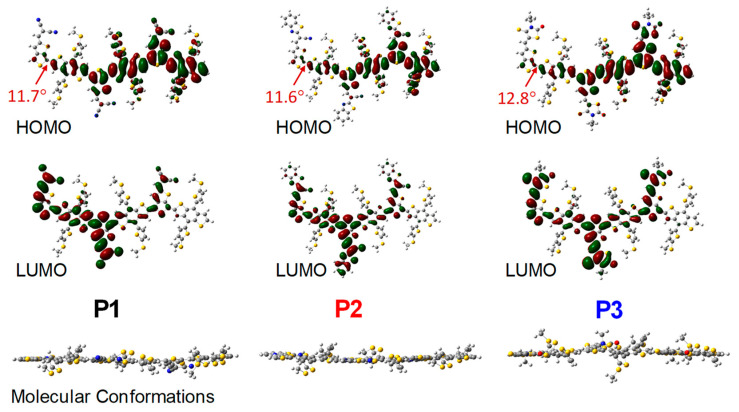
The electron density distribution of the copolymers using density function theory calculations based on the analogous trimer model and the side view molecular conformations of the copolymers.

To obtain the fundamental molecular architecture of these copolymers, theoretical calculations were performed by using Q-Chem with the B3LYP functional and 6-31G* basic set. To avoid excessive computation demand, the calculations were performed where all alkyl chain substituents were replaced with ethyl groups. Figure 4 shows the resulting geometries and the HOMO and LUMO surface plots. The HOMO state densities are nearly distributed along the polymer main chains, while those of the LUMO states are primarily localized on the thienothiophene units and acceptor groups. These results indicate that the HOMO–LUMO transition for the three copolymers is mainly accompanied by charge transfer from the electron-rich segments to the electron-poor units due to the electron deficiency of the acceptor groups on the thienothiophene units.

The dihedral angles between the BDT unit and TT unit of the polymer backbone are 11.6, 11.7 and 12.8 for P1, P2, and P3, respectively. This implies the polymer backbone is approximately coplanar, which is beneficial for efficient charge transfer, as evidenced by the ultraviolet-visible spectroscopy absorption spectra. The P3 sample showed the largest dihedral angle, suggesting that less efficient charge transfer in absorption spectra and lower HOMO energy levels would be observed in P3 as compared to others.

### 3.4. Photovoltaic Properties of PSCs

**Figure 5:**
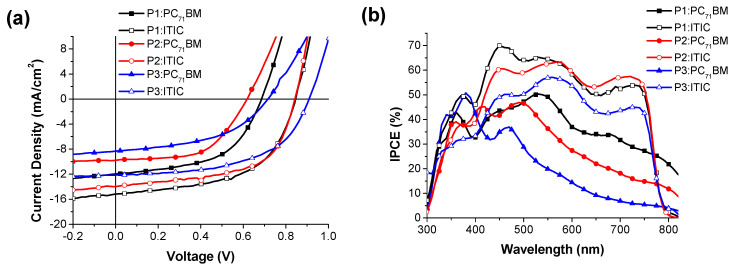
(**a**) *J*-*V* characteristics and (**b**) incident photon-to-electron conversion efficiency (IPCE) spectra of the copolymer:PC_71_BM or ITIC-based polymer solar cells (PSCs) under illumination of AM 1.5 G, 100 mW/cm^2^.

To probe the photovoltaic properties, BHJ PSCs were prepared using P1 through P3 as the donor and PC_71_BM as the acceptor with a conventional structure of indium tin oxide (ITO)/PEDOT:PSS/copolymer:PC_71_BM/Ca/Al and measured under simulated AM 1.5 G illumination (100 mW cm^−2^). The photocurrent density–voltage (*J-V*) curves of the optimal PSCs are plotted in Figure 5a, and the relevant average photovoltaic parameters including *V*_oc_, *J*_sc_, fill factor (FF), and PCE, are listed in Table 3. The optimum PCEs for the P1:PC_71_BM-, P2:PC_71_BM-, and P3:PC_71_BM-based PSCs were 4.41, 3.43, and 2.82%, respectively, with corresponding *J*_sc_ values of 11.97, 9.78, and 8.80 mA cm^−2^; *V*_oc_ values of 0.682, 0.622, and 0.702 V; and FF values of 54.09, 56.37, and 50.11%, respectively. P1 exhibited the highest *J*_sc_, which is illustrated by the broad absorption region resulting from the strong ICT interactions that led to a higher *J*_sc_. Moreover, the P1 showed the smallest dihedral angles, which is beneficial for the charge transporting and thus a higher *J*_sc_ was obtained. The highest *V*_oc_ value was obtained in the P3-based device, which is attributed to a lower HOMO energy level compared to that of the others. Although the P3-based device exhibited the highest *V*_oc_ value, but showed the lowest PCE, which is ascribed to the less efficient charge transfer in absorption spectra and morphology of blend films (discussed later).

PSCs based on these polymers coupled with the non-fullerene acceptor ITIC were also fabricated with an inverted structure of ITO/ZnO/copolymer:ITIC/MoO_3_/Ag. The *J-V* curves of the best PSC devices under the illumination of AM 1.5G at 100 mW cm^−2^ are also shown in Figure 5a, and the detailed photovoltaic characteristics are summarized in Table 3. Much higher PCEs were obtained when using the copolymer:ITIC-based PSCs. The PCEs for the P1:ITIC-, P2:ITIC-, and P3:ITIC-based PSCs were 7.25, 6.95, and 6.09%, respectively, with corresponding *V*_oc_ of 0.842, 0.841, and 0.902 V; *J*_sc_ of 15.17, 13.98, and 12.13 mA cm^−2^; and FF of 56.76, 59.09, and 55.69%, respectively. The enhanced device performance appears to come predominantly from the increased *V*_oc_ and *J*_sc_. Normally, the *V*_oc_ is primarily determined by the difference between the HOMO level of the polymer donor and the LUMO level of the acceptor (PC_71_BM or ITIC), thus the increased *V*_oc_ is ascribed to the lower LUMO energy level of ITIC (−3.83 eV) compared with that of PC_71_BM (−4.00 eV) [41,42]. Meanwhile, ITIC showed strong absorption in the long-wavelength region; therefore, the improved *J*_sc_ might result from the better complementary absorption with ITIC than with that of PC_71_BM. In addition, the lower LUMO energy levels were more offset between copolymers and ITIC than between copolymers and PC_71_BM, which is beneficial for charge transporting and leads to a higher *J*_sc_. The P1:ITIC- and P2-ITIC-based devices displayed higher PCEs than that of P3-ITIC-based device, which is ascribed to the relatively high absorption intensity in the long-wavelength region of the absorption spectra and molecular planarity of P1 and P2. The suitable LUMO energy level offsets between the copolymers and ITIC would be another reason that boosts the device efficiencies. Notably, compared with the copolymer:PC_71_BM-based PSCs, the enhancements in *J*_sc_ values were 3.20, 4.20 and 4.13 mA cm^−2^ for P1:ITIC-, P2:ITIC-, and P3:ITIC-based PSC, respectively. The large enhancements implied better complementary absorption spectra with ITIC would be beneficial for harvesting sunlight as much as required [43].

The incident photon-to-electron conversion efficiency (IPCE) spectra of the copolymer:PC_71_BM-based and copolymer:ITIC-based blend films were generated and are depicted in Figure 5b. As shown in Figure 5b, the IPCE profiles of the copolymer:PC_71_BM-based devices are consistent with the corresponding absorption spectra of the copolymers and agree well with the *J*_sc_ values obtained from the *J*-*V* measurements, indicating that the broader absorption region and higher external quantum efficiency (EQE) value contribute to the photocurrent generation and result in a higher PCE of the device. For the copolymer:ITIC-based devices, all the EQE spectra exhibited similar profiles, and a shoulder appeared at around 750 nm, which is attributed to the acceptor ITIC contribution to the photoresponse generation, implying a strong ICT occurred from the polymer donor to the ITIC acceptor. A combination of the polymer donor and ITIC acceptor yielded broad EQE spectra from 300 to 800 nm, and higher EQE values were obtained as compared to those of the copolymer:PC_71_BM-based devices, leading to higher *J*_sc_ values.

### 3.5. Analysis of Surface Morphologies

**Figure 6:**
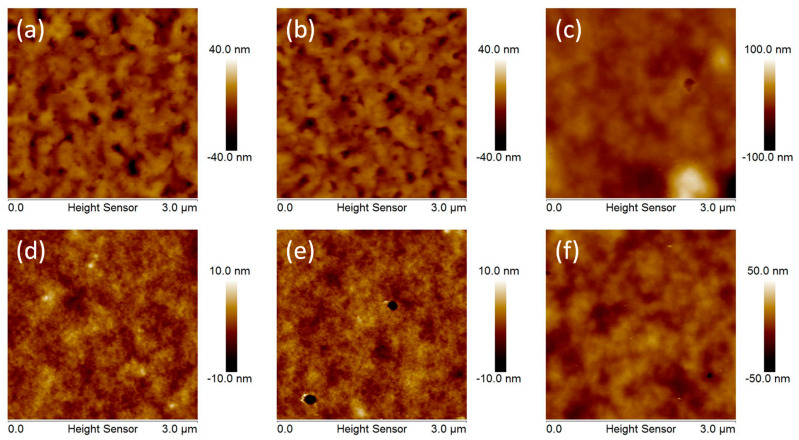
Atomic force microscopy (AFM) height images of (**a**) P1:PC_71_BM, (**b**) P2:PC_71_BM, (**c**) P3:PC_71_BM, (**d**) P1:ITIC, (**e**) P2:ITIC, and (**f**) P3:ITIC blend films.

Appreciable phase separation in a blend film is crucial to the device’s performance. Therefore, the surface morphologies of these blend films, which are identical to the active layers of the devices listed in Table 3, were studied by atomic force microscopy (AFM) and transmission electron microscopy (TEM). As shown in Figure 6a–c, the AFM images of the P1:PC_71_BM- and P2:PC_71_BM-based blend films displayed visible grains, which originated from the aggregation of PC_71_BM, whereas the P3:PC_71_BM-based blend film exhibited poor miscibility with relatively large-scale phase separation between the copolymer and PC_71_BM, which is unfavorable for charge separation. The root-mean-square (RMS) roughnesses for the P1:PC_71_BM-, P2:PC_71_BM- and P3:PC_71_BM-based blend films were 4.37, 5.06 and 9.75 nm, respectively. A relatively small RMS roughness implied better miscibility between copolymer and PC_71_BM, which is beneficial for charge separation and collection, leading to a better PCE in P1:PC_71_BM-based device [44]. The results also indicated the device performance is strongly affected by the blend film morphology, which agrees well with the results obtained from the *J-V* characteristics and IPCE results. More obvious microphase separation with appropriate domain size was observed for the copolymer:ITIC-based blend films shown in Figure 6d–f than for the copolymer:PC_71_BM blend films. This difference implied a better microphase separation would be beneficial to more effective exciton migration to the donor/acceptor interface for charge separation [45], leading to a high *J*_sc_ and FF and resulting in a higher PCE than the copolymer:PC_71_BM-based blend films. As discussed above, the improved *J*_sc_ also resulted from the better complementary absorption with ITIC than with PC_71_BM, as well as a suitable LUMO offset. The RMS roughness for the P1:ITIC-, P2: ITIC-, and P3: ITIC-based blend films were 1.14, 1.19 and, 5.33 nm, respectively. A smoother surface compared to that of the copolymer:PC_71_BM-based blend films implies better contact with the metal electrodes, which is beneficial to charge transmission and gathering. Consequently, the copolymer:ITIC-based devices exhibited higher performance than the copolymer:PC_71_BM-based devices.

**Figure 7:**
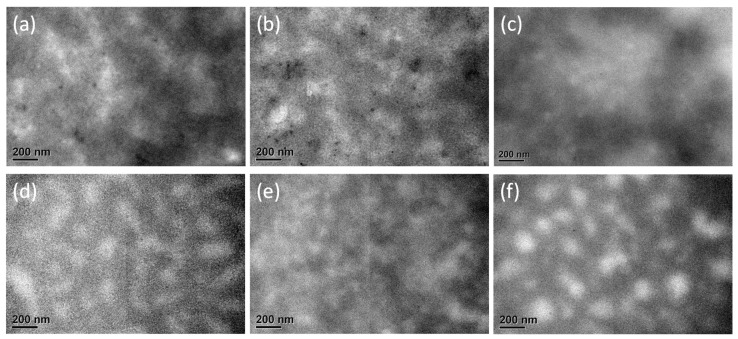
TEM images of (**a**) P1:PC_71_BM, (**b**) P2:PC_71_BM, (**c**) P3:PC_71_BM, (**d**) P1:ITIC, (**e**) P2:ITIC, and (**f**) P3:ITIC blend films.

To verify the accuracy of the AFM images, the corresponding blend films were analyzed using TEM measurements. As displayed in Figure 7a–c, the P1:PC_71_BM- and P2:PC_71_BM-based blend films presented mild microphase separation domains with clear agglomerates, similar to the results of the AFM imaging, whereas the P3:PC_71_BM-based blend film showed a phase separation with large domains, which reduced the area for charge separation and resulted in a poor PCE. For the copolymer: ITIC-based blend films, as shown in Figure 7d–f, fiber-like polymer aggregations and phase separation with appropriate feature size were obtained. These properties are beneficial for exciton dissociation and charge transport, and, as a result, lead to a higher PCE than that of the copolymer:PC_71_BM-based devices, which aligns with the results obtained from the *J-V* characteristics and IPCE results.

## 4. Conclusions

In summary, three 2D donor–acceptor conjugated copolymers consisting of a BDT derivative, TT, with a conjugated side chain attaching various acceptors as end groups were synthesized for use in PSCs. All three copolymers displayed broad absorption, ranging from 300 to 900 nm, but exhibited different ICT absorption peaks, which is ascribed to the various acceptor strengths on the TT units as well as the different dihedral angles between the BDT unit and TT unit of the polymer backbone. Similarly, the energy levels of these copolymers were also significantly affected by the acceptor strength on the TT unit and dihedral angles; therefore, P3 displayed a lower HOMO energy level than that of P1 and P2. Conventional PSCs based on copolymer:PC_71_BM in a weight ratio of 1:1.5 were fabricated and characterized, and the PCEs were estimated to be 4.41, 3.43, and 2.82% for P1, P2, and P3, respectively. Higher solar cell efficiencies were achieved when using copolymer:ITIC-based (1:1, *w*/*w*) inverted PSCs, and the PCEs were found to be 7.25%, 6.95%, and 6.05% for P1, P2, and P3, respectively. This is attributed to complementary absorption with ITIC, better morphology of the copolymer:ITIC blend films, and suitable energy level offsets between the copolymers and ITIC, leading to higher *V*_oc_, *J*_sc_, and FF, and, thus, higher PCEs. Our results imply that the conjugated side chain-end groups of the TT unit not only affect the electronic and photophysical properties of the copolymers but also influence the morphologies of the blend films and the performance of the overall device. With successfully building up the copolymer skeleton, further copolymers attaching different electron-withdrawing end groups with improved device efficiency will be investigated in the near future.

## Figures and Tables

**Table 1:** Molecular weights and thermal properties of copolymers.

Copolymers	M_n_^a^ (g/mol)	M_w_^a^ (g/mol)	PDI (M_w_/M_n_)	*T*_d_^b^ (°C)
**P1**	16,250	36,800	1.65	285
**P2**	21,000	25,900	1.23	314
**P3**	12,820	18,200	1.42	321

^a^ M_n_, M_w_ and polydispersity index (PDI) of the polymers were determined by gel permeation chromatography (GPC) using polystyrene standards in tetrahydrofuran (THF). ^b^ Temperature of 5% weight loss.

**Table 2:** Photophysical and electrochemical properties of copolymers.

Copolymers	In Solution *^a^* (nm)	In Film *^b^* (nm)	HOMO/LUMO *^c^* (eV)	$E_{g}^{ec}$^*d*^ (V)
	$\lambda_{max}^{abs}$	$\lambda_{max}^{abs}$		
**P1**	355, 518, 717	359, 528, 718	−5.50/−3.69	1.81
**P2**	382, 518, 704	381, 524, 707	−5.54/−3.65	1.89
**P3**	373, 539	363, 587	−5.60/−3.72	1.88

*^a^* Measured in *o*-dichlorobenzene solution. *^b^* Casted from *o*-dichlorobenzene solution. *^c^* The energy levels were calculated according to: HOMO = −e($E_{on}^{ox}$ − $E_{on}^{ferrocene}$ + 4.8) (eV), LUMO = −e($E_{on}^{re}$ − $E_{on}^{ferrocene}$ + 4.8) (eV). *^d^* Estimated using empirical equations: $E_{g}^{ec}$ = $E_{HOMO}$–$E_{HOMO}$.

**Table 3:** Photovoltaic performances of the bulk heterojunction solar cells.

Active Layer	*V_oc_* (V)	*J_sc_* (mA/cm^2^)	FF (%)	PCE (%)	Thickness (nm)
**P1:PC_71_BM**	0.682 ± 0.009	11.97 ± 0.15	54.09 ± 0.17	4.41 ± 0.24	120
**P1:ITIC**	0.842 ± 0.008	15.17 ± 0.17	56.76 ± 0.15	7.25 ± 0.31	115
**P2:PC_71_BM**	0.622 ± 0.009	9.78 ± 0.11	56.37 ± 0.12	3.43 ± 0.18	110
**P2:ITIC**	0.841 ± 0.009	13.98 ± 0.22	59.09 ± 0.19	6.95 ± 0.40	120
**P3:PC_71_BM**	0.702 ± 0.009	8.00 ± 0.10	50.11 ± 0.11	2.82 ± 0.15	125
**P3:ITIC**	0.902 ± 0.010	12.13 ± 0.19	55.69 ± 0.21	6.09 ± 0.37	108
